# Improving the Timeliness of Discharge Summaries in Acute Inpatient Psychiatric Wards: A Quality Improvement Project

**DOI:** 10.1192/bjo.2026.11474

**Published:** 2026-06-30

**Authors:** Ozgun Ekin Sahin, Zeynepgul Kalay

**Affiliations:** 1 Oxleas NHS Foundation Trust, London, United Kingdom; 2 South London and Maudsley NHS Foundation Trust, London, United Kingdom

## Abstract

**Aims:**

The aim of the QIP is to reduce the average time between patient discharge and discharge summary completion from the current baseline (10–15 days) to within 7 days post discharge for more than 70% of discharges on Norman and Betts Ward.

We tried to achieve this by ensuring resident doctors have protected time to complete discharge summaries, providing a structured and standardized discharge summary template to improve consistency, and raising awareness among staff regarding the importance of timely discharge documentation to enhance communication with GPs and Community Mental Health Teams.

**Methods:**

Baseline data were collected retrospectively between March and June 2025 on the Norman and April and July on Betts acute inpatient psychiatric wards, measuring the timebetween patient discharge and discharge summary completion. Post intervention data were collected prospectively over five months following the introduction of protected time for resident doctors.

An intervention was implemented to improve discharge planning, including protected time for resident doctors dedicated to discharge summaries, and a standardised discharge summary template.

The intervention began in July 2025 on Norman Ward and in August 2025 on Betts Ward, with data collected prospectively as the changes were implemented. The primary outcome measured was the proportion of discharge summaries completed within 7 days of patient discharge. Secondary outcomes included mean completion time of discharge summaries.

Data were analysed using SPSS with appropriate comparative tests. Informal staff feedback was collected to assess acceptability. The project was registered with Oxleas NHS Foundation Trust.

**Results:**

During the post-intervention period, a total of 95 patients were discharged from both wards. Both wards demonstrated a significant reduction in discharge summary completion times. Mean completion time decreased from 11.3 to 3.7 days on Norman Ward and from 15.4 to 6.2 days on Betts Ward. Mann–Whitney U test showed a statistically significant reduction in completion time for both wards (p <0.001).

Post intervention, 80% of discharge summaries on Norman Ward and 66% on Betts Ward were completed within 7 days, compared with 31% across both wards at baseline, demonstrating a marked overall improvement.

**Conclusion:**

After the intervention discharge summary completion times significantlyreduced and increased the proportion completed within 7 days. The intervention is feasible, effective, and sustainable, with plans to initiate protected discharge summary time as routine practice and handover processes to future doctors to maintain continuity and patient safety.

